# The clock is ticking: the rate and timeliness of antiretroviral therapy initiation from the time of treatment eligibility in Kenya

**DOI:** 10.7448/IAS.18.1.20019

**Published:** 2015-10-26

**Authors:** Thomas A Odeny, Brendan DeCenso, Emily Dansereau, Anne Gasasira, Caroline Kisia, Pamela Njuguna, Annie Haakenstad, Emmanuela Gakidou, Herbert C Duber

**Affiliations:** 1 Institute for Health Metrics and Evaluation, University of Washington Seattle, WA, USA; 2 Kenya Medical Research Institute, Nairobi, Kenya; 3 RTI International, Research Triangle Park, NC, USA; 4 African Leaders Malaria Alliance, Kampala, Uganda; 5 Africa Action Help-International, Nairobi, Kenya; 6 Afya Resource Associates, Nairobi, Kenya

**Keywords:** HIV, antiretroviral therapy, ART initiation, Africa, Kenya, time to start treatment

## Abstract

**Introduction:**

Understanding the determinants of timely antiretroviral therapy (ART) initiation is useful for HIV programmes intent on developing models of care that reduce delays in treatment initiation while maintaining a high quality of care. We analysed patient- and facility-level determinants of time to ART initiation among patients who initiated ART in Kenya.

**Methods:**

We collected facility-level information and conducted a retrospective chart review of adults initiating ART between 2007 and 2012 at 51 health facilities in Kenya. We evaluated the association between patient- and facility-level covariates at the time of ART eligibility and time to ART initiation. We also explored the determinants associated with timeliness of ART initiation.

**Results:**

The analysis included 11,942 patients. The median age at the time eligibility was first determined was 37 years (interquartile range [IQR] 31–45). Overall, 75% of patients initiated ART within two months of eligibility. The median CD4 cell count at the time eligibility was first determined rose from 132 (IQR 51–217) in 2007 to 195 (IQR 91–286) in 2011 to 2012 (*p*<0.001). The cumulative probability of ART initiation among treatment-eligible patients increased over time: 87.1% (95% confidence interval [CI] 85.1–89.0%) in 2007; 96.8% (96.0–97.5%) in 2008; 97.1% (96.3–97.7%) in 2009; 98.5% (98.0 −98.9%) in 2010; and 99.7% (95% CI 99.4 −99.8%) in 2011 to 2012 (*p<*0.0001). In multivariate analyses, attending a health facility with high ART patient volumes within two months of eligibility was considered the key facility-level determinant of ART initiation (adjusted odds ratio 0.57, 95% CI 0.45–0.72, *p<*0.001). Patient-level determinants included being eligible for ART in the years subsequent to 2007, advanced World Health Organization clinical stage and low CD4 cell count at the time eligibility was first determined.

**Conclusions:**

Overall, the time between treatment eligibility and ART initiation decreased substantially in Kenya between 2007 and 2012, with uniform gains across different types of health facilities. Our findings highlight the slow increase in CD4 cell counts at the time of ART eligibility over time, indicating that a large number of patients are still beginning ART with advanced HIV disease. Our findings also support the decentralisation of ART services at all health facilities that have the capacity to initiate treatment. Continued evaluation of programme- and country-level data is needed to monitor timeliness of ART initiation as countries continue to expand treatment access.

## Introduction

HIV/AIDS is a leading cause of disease burden in sub-Saharan Africa [1,2]. Timely initiation of antiretroviral therapy (ART) is an important component of the HIV care cascade. Delays between ascertainment of ART eligibility and treatment initiation are associated with higher risk of mortality compared with initiation of ART without delay [3–5]. Pre-treatment mortality among patients eligible for ART has been documented between 30 deaths per 100 person-years in South Africa [6,7] and about 35 deaths per 100 person-years in East Africa [8]. These rates are likely underestimates since they do not incorporate outcomes for patients lost to follow-up between eligibility and treatment initiation [9]. Failure to initiate ART among treatment-eligible patients is also associated with substantial losses to follow-up prior to treatment initiation [8,10,11].

Causes of delayed treatment initiation are numerous and varied. Many patients delay ART initiation as they await adherence counselling [12], yet such counselling is not associated with better adherence or other health outcomes [13,14]. For less clear reasons, the plateauing of donor funding for public HIV programmes in sub-Saharan Africa has been associated with delays in ART initiation [15]. Recent World Health Organization (WHO) guidelines expanding recommendations for initiating ART at higher CD4 cell counts have resulted in increasing demand for treatment in HIV programmes, yet a large number of eligible patients are still awaiting initiation [16]. Therefore, as HIV programmes in sub-Saharan Africa expand rapidly, monitoring of timely ART initiation might become an important indicator of the quality of care in ART programmes.

Clinical characteristics associated with mortality during the time between ART eligibility and initiation have been studied before [7,9,17–20]. However, there is a paucity of literature regarding patient- and clinic-level characteristics associated with delayed initiation of ART among treatment-eligible patients. Understanding the determinants of timely ART initiation might be useful for HIV programmes intent on developing models of care that reduce delays in treatment initiation while maintaining a high quality of care. We aimed to analyse patient- and facility-level determinants of time to ART initiation among patients who initiated ART in Kenya.

## Methods

### Study population

As part of a larger facility-based costing study (Access, Bottlenecks, Costs, and Equity) [21], a health facility survey was conducted in a nationally representative sample of 254 health facilities in Kenya between June and November 2012. Information was collected across a wide array of categories, including finances, human resources, equipment, pharmaceuticals, and outputs. At 60 of these facilities that also delivered ART, clinical records were extracted for adult patients (age ≥18 years) who had initiated ART between six and 60 months prior to the date of record extraction [22].

At each of the designated ART facilities that agreed to patient chart extraction, research assistants asked to see all patient records, specifically requesting charts of deceased patients and those lost to follow-up in addition to patients currently enrolled in treatment. Once all available and eligible patient records were identified, either the research assistant estimated the total number of charts with the help of the facility administrator or the number of charts, if known, was recorded. In facilities in which there were fewer than 250 charts, all charts were extracted. If a facility had more than 250 charts, the total number of charts was divided by 250, and then using the closest integer (*x*) to that result, every *x* chart was selected for record extraction until 250 charts were reviewed. Four facilities provided a complete set of electronic medical records. Nine facilities with fewer than 50 patient records were excluded from this analysis.

We assumed that eligibility for ART was determined at each facility according to the Kenyan national guidelines for ART [23]. Prior to August 2010, patients were eligible for ART if they had (1) CD4 cell count less than 200 cells/µL, where CD4 testing was available, and WHO stage I or II clinical disease; (2) CD4 cell count less than 350 cells/µL, and WHO stage III clinical disease; or (3) WHO stage IV clinical disease, irrespective of CD4 cell count. In August 2010, patients became eligible for ART if they had a CD4 cell count of less than 350 cells/µL or WHO stage III or IV clinical disease, irrespective of CD4 cell count.

Patient- and facility-level variables for inclusion in analyses were based on *a priori* selection because of being known or suspected potential determinants of time to ART initiation. All facility-level variables were binary indicators. Specifically, health facility platforms were categorised as (1) either hospitals (sub-district, district, provincial and national referral hospitals) or health centres (health centres and medical clinics); (2) ART clinic size was a facility- and year-level covariate categorised as either above or below the all-facility median number of ART patient volumes in a patient's year of initiation; (3) facility location was either urban (including peri-urban) or rural; (4) facility ownership was either public (owned and run by the government) or private (faith based, non-government organisations, private); (5) receipt of bonuses for ART staff (responses to “Do the ART staff receive bonuses or top ups at this facility?”); (6) availability of outreach services (responses to “Does this facility offer outreach services?”); (7) physician versus nurse leadership (responses to “Who leads the care related to HIV/AIDS at this facility?”); and (8) availability of HIV treatment guidelines (responses to “Indicate whether or not the following guidelines are present. A guideline can be a document, poster, etc.; they need not be posted but should be made available to the medical personnel. If you do not get to see them, inquire with a medical personnel if there are any of the following guidelines available, Disease-specific treatment guidelines: HIV”) were all categorised as either *available* or *unavailable*.

Patient-level characteristics included age, sex, year of eligibility for ART, CD4 cell count, and WHO clinical stage at the time eligibility was first determined. Where date of ART initiation was not documented in the chart, we used the earliest visit date when a treatment regimen was indicated in the chart. Data on CD4 cell count and WHO clinical stage at the time eligibility was first determined were not documented for a large proportion of patients [24], as has been the case in other similar cohorts [25,26]. To determine the association between these important covariates and ART initiation, we included comparison categories for missing CD4 cell count and WHO clinical stage. CD4 cell counts were therefore categorised as ≤50, 51 to 200, 201 to 350, >350, and missing. Year of ART eligibility was categorised as 2007, 2008, 2009, 2010, and 2011 to 2012.

### Analysis

We focused our analysis on three aspects related to timely ART initiation, with the patient as the unit of analysis. First, to compare the time from ART eligibility to initiation, we used Kaplan-Meier survival curves and log-rank tests to estimate the cumulative probability of ART initiation from the time eligibility was first determined. The dates of ART initiation available from the records included only the month and year of initiation. Therefore, we assumed that initiations occurred evenly throughout the month and used the middle of each month as the date of initiation. Second, to evaluate the association between baseline covariates (at the time eligibility was first determined) and time to ART initiation, we used a multilevel Cox proportional hazards regression model with shared frailty to account for unobserved heterogeneity between patients at different health facilities. We assessed the proportional hazards assumption using tests and graphs based on scaled Schoenfeld residuals. Year of eligibility did not satisfy the proportional hazards assumption and was modelled as a time-varying covariate. To explore the determinants associated with timely ART initiation (defined as receiving ART within two months of eligibility for treatment), we used a logistic regression model with a random intercept for the health facility and included the same covariates as in the previous Cox proportional hazards model. Results are presented separately for patient- and facility-level determinants. We used Stata/SE v12.1 (StataCorp, College Station, TX, USA) for all analyses.

### Ethical considerations

Ethical approval was obtained from the University of Washington Human Subjects Division and the Kenya Medical Research Institute Ethical Review Committee.

## Results

### Characteristics at ART eligibility

Most facilities were classified as public (90%), were located in urban areas (71%), did not pay bonuses for ART staff (90%), provided outreach services (78%) and reported being led by a physician (75%) (Table 1). We extracted charts for 15,753 patients. We excluded 3,520 patients who did not have a recorded date of ART eligibility and whose historical CD4 cell count and WHO staging records were inadequate to determine eligibility. Records for an additional 291 patients were excluded due to lack of information about the date of ART initiation. All of the remaining 11,942 patients were included in the analysis. Patients whose records did not indicate a date of ART eligibility or initiation had higher proportions of missing information about other patient characteristics such as age, sex, year of ART eligibility, WHO clinical stage, and CD4 cell count (Table 2). Baseline facility-level characteristics among patients with eligibility and initiation dates versus those without are given in the Supplementary file.

**Table 1:** Characteristics of the 51 health facilities included in the analysis

	*N*	%
Management		
Public	46	90.2
Private	5	9.8
Location		
Urban	36	70.6
Rural	15	29.4
Bonuses for ART staff		
No	46	90.2
Yes	3	5.9
Not recorded	2	3.9
Outreach services		
None	11	21.6
Available	40	78.4
Leadership		
Nurse	11	21.6
Physician	38	74.5
Not recorded	2	3.9
Platform		
Health centre	26	51.0
Hospital	25	49.0
ART clinic size		
Below median	23	45.1
Above median	26	51.0
Not recorded	2	3.9
HIV treatment guidelines		
Not present	2	3.9
Present	49	96.1

ART, antiretroviral therapy.

**Table 2:** Characteristics at the time eligibility for antiretroviral therapy initiation was first determined (baseline) comparing patients with eligibility and initiation dates versus those without

	Eligibility and initiation dates reported	Eligibility and initiation dates missing	
			
	(*N=*11,942)	(*N=*3,811)	
			
	*N* (%)	*N* (%)	Chi-square *p*-value
Sex			
Male	4,150 (34.8)	1,156 (30.3)	<0.001
Female	7,762 (65.0)	2,619 (68.7)	
Not recorded	30 (0.3)	36 (0.9)	
Age (years)			
<30	2,363 (19.8)	49 (1.3)	<0.001
30 to 39	4,605 (38.6)	109 (2.9)	
40 to 49	3,169 (26.5)	64 (1.7)	
≥50	1,768 (14.8)	55 (1.4)	
Not recorded	37 (0.3)	3,534 (92.7)	
Year of ART eligibility			
2007	1,095 (9.2)	9 (0.2)	<0.001
2008	2,168 (18.2)	45 (1.2)	
2009	2,257 (18.9)	45 (1.2)	
2010	2,857 (23.9)	68 (1.8)	
2011 to 2012	3,565 (29.9)	133 (3.5)	
Not recorded	(0)	3,511 (92.1)	
WHO clinical stage			
I	1,359 (11.4)	180 (4.7)	<0.001
II	2,789 (23.4)	367 (9.6)	
III	4,509 (37.8)	542 (14.2)	
IV	448 (3.8)	105 (2.8)	
Not recorded	2,837 (23.8)	2,617 (68.7)	
CD4 cell count (cells/µL)			
≤50	1,739 (14.6)	203 (5.3)	<0.001
50 to 200	3,963 (33.2)	468 (12.3)	
201 to 350	3,124 (26.2)	416 (10.9)	
>350	369 (3.1)	256 (6.7)	
Not recorded	2,747 (23)	2,468 (64.8)	

The median number of patient charts extracted at each facility was 153 (interquartile range [IQR] 71–211). The median age at the time eligibility was first determined was 37 years (IQR 31–45), 7,762 (65%) patients were female and 6,422 (54%) were eligible for treatment initiation between 2010 and 2012. Among the 9,195 (77%) patients with a CD4 cell count recorded, the median CD4 cell count at the time eligibility was first determined was 163 cells/µL (IQR 71–244). Of the 9,105 (76%) patients with a record for WHO stage, 4,957 (54%) had advanced clinical HIV disease at the time eligibility was first determined (WHO stage III or IV).

Patients treated at hospitals had the same median age at ART eligibility (37 years, IQR 31–45) as those at health centres (37 years, IQR 31–44), but had a lower median CD4 cell count (159 cells/µL, IQR 65–243) compared with those at health centres (172 cells/µL, IQR 86–248). Over time, there was a significant upward trend in the median CD4 cell count (measured at the first documented assessment for treatment eligibility), increasing from 132 cells/µL (IQR 51–217) in 2007 to 195 cells/µL (IQR 91–286) in 2011 to 2012 (*p*<0.001). The proportion of patients with advanced clinical disease at the time eligibility was first determined was similar at hospitals (54%) and health centres (56%) (*p=*0.1) (data not shown). There was no significant trend in the proportion of individuals with advanced clinical disease over time: 56% in 2007, 62% in 2008, 60% in 2009, 53% in 2010, and 48% in 2011 to 2012.

### Time to ART initiation

**Figure 1:**
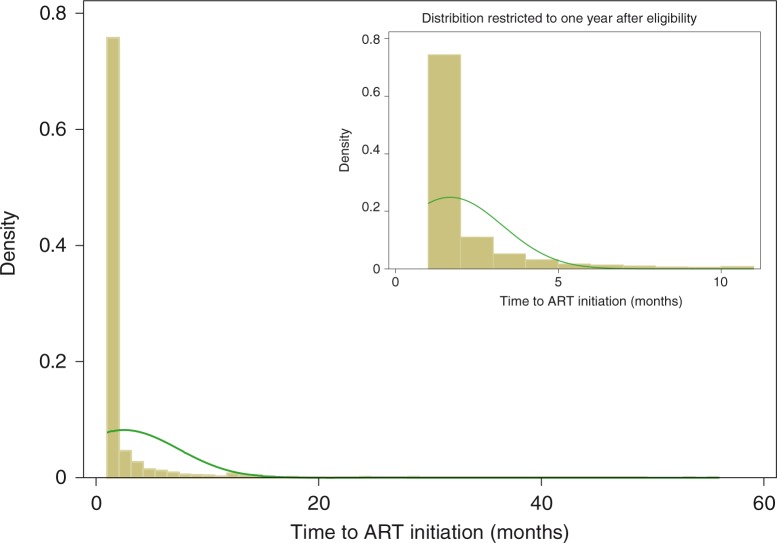
Histogram showing distribution of time to antiretroviral therapy initiation.

**Figure 2:**
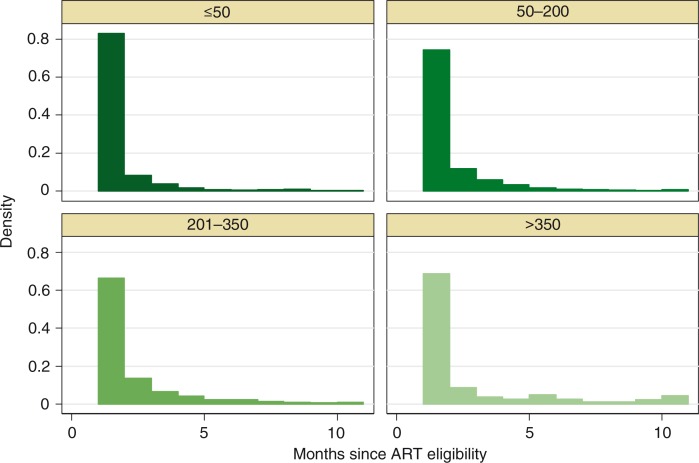
Distribution of time to antiretroviral therapy initiation within the first year after eligibility stratified by categories of CD4 cell count.

Overall, 75% of patients initiated ART within two months of eligibility (Figure 1). The median time to initiation was one month. The distribution of time to ART initiation (within the first year after eligibility) was similar when stratified by categories of CD4 cell count (Figure 2).

**Figure 3:**
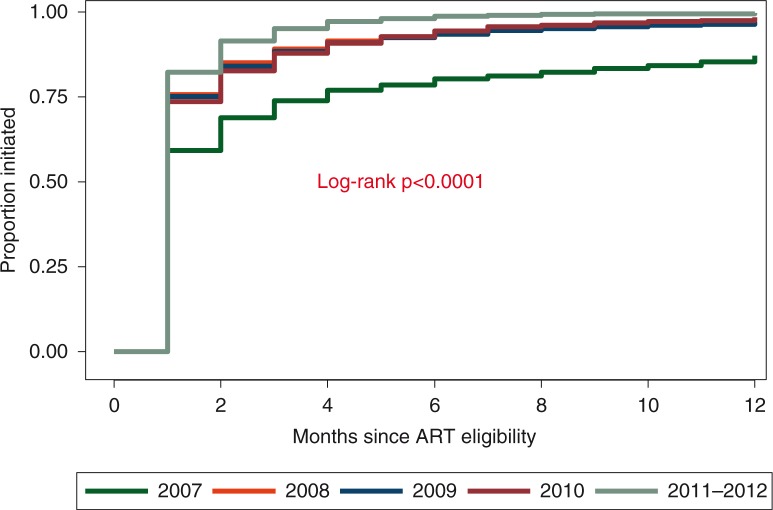
Kaplan-Meier plot of time from antiretroviral therapy eligibility to initiation by year of eligibility.

At 1, 12, and 24 months after ART eligibility, the cumulative probabilities of ART initiation among treatment-eligible patients were 75.5% (95% confidence interval [CI] 74.8–76.3%), 97.2% (95% CI 96.9–97.5%) and 99.1% (95% CI 98.9–99.2%), respectively. When restricted to the first year after eligibility, the cumulative probability of ART initiation increased significantly each year from 87.1% (95% CI 85.1–89.0%) in 2007 to 99.7% (95% CI 99.4–99.8%) in 2011 to 2012 (*p<*0.0001) (Figure 3).

### Determinants of the rate of ART initiation

Patient- and facility-level determinants of the rate of ART initiation are summarised in Table 3, Column 1. Patient gender, age and WHO clinical stage were not significantly associated with the rate of ART initiation. In contrast, patients eligible for ART after 2007 had significantly higher rates of initiation compared with those eligible in 2007. Compared with patients with CD4 cell counts higher than 350 cells/µL, patients with CD4 cell counts lower than 200 cells/µL had significantly higher rates of ART initiation, as did patients without a documented CD4 cell count. Patients with baseline CD4 cell counts between 201 and 350 cells/µL did not differ in the rates of initiation when compared with those with CD4 cell counts greater than 350 cells/µL.

**Table 3:** Patient and facility-level determinants of (1) time to ART initiation and (2) timely ART initiation in the first two months after eligibility

	(1) Time to ART initiation Cox proportional hazard model		(2) ART initiation within two months Logistic regression model	
		
	Hazard ratio (95% CI)	*p*	Odds ratio (95% CI)	*p*
*Patient characteristics*				
Female	0.97 (0.93–1.01)	0.15	0.91 (0.82–1.00)	0.06
Age in years (reference: <30)				
30 to 39	1.05 (1.00–1.10)	0.07	1.08 (0.95–1.22)	0.22
40 to 49	1.04 (0.98–1.10)	0.16	1.06 (0.93–1.22)	0.37
≥50	1.06 (0.99–1.13)	0.08	1.10 (0.94–1.29)	0.24
Year of ART eligibility (reference: 2007)				
2008	1.32 (1.22–1.43)	<0.001	2.41 (2.04–2.84)	<0.001
2009	1.26 (1.16–1.37)	<0.001	2.48 (2.09–2.93)	<0.001
2010	1.25 (1.14–1.36)	<0.001	2.32 (1.97–2.74)	<0.001
2011 to 2012	1.42 (1.30–1.55)	<0.001	4.01 (3.38–4.77)	<0.001
WHO clinical stage (reference: I)				
II	1.01 (0.94–1.08)	0.74	1.02 (0.86–1.21)	0.84
III	0.94 (0.88–1.00)	0.05	0.76 (0.65–0.90)	<0.01
IV	1.03 (0.92–1.15)	0.65	1.24 (0.92–1.67)	0.16
Not recorded	1.01 (0.94–1.08)	0.73	1.09 (0.91–1.29)	0.35
CD4 cell count (cells/µL) (reference: >350)				
≤50	1.38 (1.23–1.55)	<0.001	2.88 (2.21–3.75)	<0.001
50 to 200	1.28 (1.14–1.43)	<0.001	1.75 (1.37–2.23)	<0.001
201 to 350	1.11 (0.99–1.24)	0.08	1.07 (0.83–1.37)	0.60
Not recorded	1.31 (1.17–1.46)	<0.001	2.82 (2.19–3.64)	<0.001
*Facility characteristics*				
Hospital (reference: health centre)	1.01 (0.91–1.11)	0.91	1.05 (0.56–1.96)	0.88
Rural location (reference: urban)	0.93 (0.83–1.04)	0.19	0.56 (0.28–1.13)	0.11
Public management (reference: private)	1.11 (0.95–1.30)	0.19	1.43 (0.59–3.45)	0.42
ART clinic size above median	0.94 (0.88–1.01)	0.10	0.57 (0.45–0.72)	<0.001
ART staff receive bonuses	0.89 (0.75–1.06)	0.18	0.92 (0.34–2.44)	0.86
Facility offers outreach services	1.04 (0.96–1.13)	0.33	1.22 (0.69–2.13)	0.49
Physician leadership	1.00 (0.92–1.09)	0.98	1.03 (0.59–1.79)	0.91
HIV treatment guidelines present	1.17 (1.00–1.38)	0.05	2.21 (0.77–6.33)	0.14

ART, antiretroviral therapy; CI, confidence interval.

Patients at facilities with HIV treatment guidelines available experienced higher rates of initiation compared with those at facilities without guidelines (adjusted hazard ratio 1.17, 95% CI 1.00–1.38, *p=*0.05). No other facility-level covariates were found to be significantly associated with rates of ART initiation (Table 3).

### Timely ART initiation (within two months of eligibility)

Overall, 9,019 of 11,942 ART-eligible patients (76%) initiated treatment within two months of eligibility. The results of multivariable analysis of determinants of timely ART initiation are presented in Table 3, Column 2. We found that gender, age and WHO clinical stage were not significant predictors of timely ART initiation. We also found that more recent year of eligibility, CD4 cell counts of ≤200 cells/µL and absence of a CD4 cell count record were associated with significantly higher likelihood of timely ART initiation (Table 3). Above-median patient volume was associated with a significantly lower likelihood of timely ART initiation (adjusted odds ratio 0.57, 95% CI 0.45–0.72, *p<*0.001). Other facility-level characteristics were not significantly associated with the likelihood of early ART initiation.

## Discussion

Overall, we found that the time between patient eligibility for ART and actual ART initiation decreased substantially in Kenya between 2007 and 2012, with uniform gains across different types of health facilities. This increase in initiation rates occurred against the backdrop of a dramatic scale-up in HIV programmes that resulted in increasing numbers of patients becoming eligible for ART, with 83% of eligible adults receiving ART overall [27]. In our study, we observed that a high proportion of eligible patients (76%) were started on ART within two months of being found eligible. This large fraction of patients started on treatment in a timely manner is higher than that reported for similar patients in Uganda [9]. While the increase in median CD4 cell count at ART eligibility showed a statistically significant upward trend from 2007 to 2011 through 2012, the rate of increase was slow. Similar findings were observed by Lahuerta and colleagues [28] in a recent paper where they documented advanced HIV disease at both entry into HIV care and at the time of ART initiation in four countries, including Kenya. This slow rate is concerning and might indicate that, even with the cut-off for ART initiation being raised to 500 cells/µL in Kenya, patients might still be entering HIV care with advanced disease. We found that ART clinic size was an important determinant of the likelihood of ART initiation. Patients receiving care at facilities with above-median ART patient volumes were less likely to start ART within two months of eligibility, compared with those at facilities with below-median patient volumes. This may be an indication that the increase in the number of patients receiving ART has not been accompanied by increases in the number of providers to meet the demand for initiation. This may also have implications for future treatment expansion, given that the WHO recently released new guidelines recommending ART initiation at CD4 cell counts of below 500 cells/µL [29]. The mechanisms by which higher volume facilities could result in lower rates of initiation might also include lower staff-to-patient ratios as observed in a South African cohort, heavier workloads, and longer waiting times at higher volume facilities [5,28].

Importantly, other facility-level factors were not significant determinants of either the rate or timeliness of ART initiation. For example, patients at health centres had similar rates of ART initiation as those at hospitals. Similarly, patients at public facilities had similar outcomes to those at private facilities. This information is encouraging for national HIV programmes, which will increasingly have to rely on local funding mechanisms to sustain the rapid expansion and continued decentralisation of HIV treatment.

We found that patient-level characteristics were not associated with either the rate or timeliness of ART initiation among treatment-eligible patients, consistent with other studies in East Africa [15]. We also found that patients with low CD4 cell counts (≤200 cells/µL) experienced higher rates and likelihood of initiation compared with those with CD4 levels above 350 cells/µL, consistent with results from other sub-Saharan African countries [9,15]. These findings are important as HIV programmes consider moving towards treatment as prevention strategies, in which treatment eligibility will be set at high CD4 levels [29] or where ART will be offered to all patients regardless of CD4 cell count, a trend currently being investigated in East Africa [30] and already in place in high-income countries [31].

Our study was limited in several ways. First, we restricted analysis to only patients who eventually started ART, as our data collection protocol targeted patients currently receiving ART. An analysis of all patients determined to be eligible for ART (regardless of whether they eventually started ART) would potentially provide additional useful information about factors associated with the rate and timeliness of ART initiation. However, our study provides valid information about factors associated with delays in ART initiation among patients who eventually receive treatment. Second, a large proportion of patients were missing information about CD4 cell count (23%) and WHO stage (24%) at the time eligibility was first determined. We categorised these as missing and compared their outcomes with those with information present. We believe that such a comparison still provides useful information for policymakers, since many programmes are yet to achieve universal CD4 testing, and many clinics in East Africa have incomplete records of WHO staging information [24]. Importantly, the use of missing value dummy variables could result in biased estimates. In our case, however, the inclusion of missing variables enabled us to make use of all available information that would be useful for policymakers. This inclusion of patients with unavailable information also enables comparison with other literature [26]. Third, we did not have information about tuberculosis status at the time eligibility was first determined or the presence of opportunistic infections such as cryptococcal meningitis, which are known to influence clinical decisions about the timing of ART initiation [32,33]. However, these clinical conditions are captured as part of WHO clinical staging criteria, which we included in our models. Fourth, our dataset did not have complete information about pregnancy status at the time eligibility was first determined. According to the most recent WHO guidelines, HIV programmes are encouraged to initiate all HIV-positive pregnant women on lifelong ART where feasible [29] (referred to as *WHO option B*+). It would be useful to investigate the association of pregnancy status and the rate of ART initiation to form a baseline that could allow programmes to monitor progress with implementation of the new guidelines going forward. Fifth, outreach services can vary enormously depending on the context in which they are implemented. This broader analysis has therefore highlighted specific areas that need to be investigated to provide a more granular view of the nature and types of services provided at the facility level and their effect on patient outcomes. Sixth, in August 2010 there was a change in the Kenyan guidelines for ART initiation from a CD4 cell count of 200 cells/µL to 350 cells/µL, yet our analysis was not stratified by these different eligibility criteria. Because these guidelines were implemented at the facility level at different times, we assumed that clinicians applied the prevailing treatment guidelines whenever they determined that a patient was eligible for ART initiation. Finally, other programme-level factors that might be associated with the rate and timeliness of ART initiation – such as distance to facility, number of ART staff, number of days ART clinic is open, availability of CD4 testing onsite and presence of prevention of mother-to-child transmission of HIV services – were missing and were therefore not included in the models. An important strength is that we used two different analytical methods that yielded broadly consistent results – where statistical significance varied between methods, we found that directionality was consistent. Despite these limitations, our study provides evidence of significant improvements in the rates and timeliness of ART initiation over time and identifies important determinants of ART initiation.

## Conclusions

To our knowledge, this is the first study in Kenya to assess the rates and timeliness of ART initiation at health facilities spread out across various regions and representing all levels of healthcare provision. In summary, we find that apart from patient volumes, clinic-level factors are not associated with delays in ART initiation. These findings support the decentralisation of ART services at all health facilities that have the capacity to initiate treatment, regardless of facility-level characteristics. The most recent WHO guidelines for ART lower the threshold for initiation and will substantially increase the number of treatment-eligible patients. Continued evaluation of programme- and country-level data is needed to monitor timeliness of ART initiation as countries begin to adopt and implement these new guidelines.

## Supplementary Material

The clock is ticking: the rate and timeliness of antiretroviral therapy initiation from the time of treatment eligibility in Kenya
