# Supplementary material for: The clock is ticking: the rate and timeliness of antiretroviral therapy initiation from the time of treatment eligibility in Kenya
Source: J Int AIDS Soc. 2015 Oct 26;18(1):20019. doi: 10.7448/IAS.18.1.20019 (PMC4623278; doi:10.7448/IAS.18.1.20019)
Supplement: The clock is ticking: the rate and timeliness of antiretroviral therapy initiation from the time of treatment eligibility in Kenya [file JIAS-18-20019-s001.pdf]

Additional file 1: Facility-level characteristics comparing patients with eligibility and initiation dates versus those without

	Eligibility and initiation dates reported (N=11,942) %	Eligibility and initiation dates missing (N=3,811) %	Chi-square p-value
Platform			
Health Center	28.3%	26.8%	0.058
Hospital	71.7%	73.2%	
ART clinic size			
Below median	24.0%	16.8%	<0.001
Above median	74.8%	76.9%	
Not recorded	1.2%	6.4%	
Location			
Urban	86.9%	86.6%	0.631
Rural	13.1%	13.4%	
Management			
Private	3.3%	4.2%	0.006
Public	96.7%	95.8%	
Bonuses for ART staff			
No	95.7%	96.9%	<0.001
Yes	2.1%	2.3%	
Not recorded	2.1%	0.8%	
Outreach services			
None	17.8%	10.9%	<0.001
Available	82.2%	89.1%	
Leadership			
Nurse	26.1%	34.7%	<0.001
Physician	72.0%	64.5%	
Not recorded	1.9%	0.8%	
HIV treatment guidelines			
Not present	2.9%	0.9%	<0.001
Present	97.1%	99.1%	
